# Severe Dilatation of Coronary Artery Ostium Complicating Sinus of Valsalva Aneurysm: Differential Diagnosis and Review of the Literature

**DOI:** 10.1155/2017/8694652

**Published:** 2017-04-13

**Authors:** Paul Bamford, Nicholas Collins

**Affiliations:** John Hunter Hospital, Newcastle, NSW 2305, Australia

## Abstract

Coronary artery dilatation may be due to various aetiologies including congenital anomalies, atherosclerotic coronary disease, and Kawasakis disease. We describe a case characterised by apparent severe dilatation of the right coronary artery ostium in an asymptomatic male. Subsequent imaging and surgical intervention documented the presence of a sinus of Valsalva aneurysm extending into the ostium of the right coronary artery. This represents an unusual manifestation of a sinus of Valsalva aneurysm. The underlying pathophysiology, differential diagnosis, role of surgical management, and outcomes are discussed.

## 1. Introduction

The aortic sinuses of Valsalva are areas of dilatation of the aortic root that arise from the three cusps of the aortic valve [1]. Sinus of Valsalva aneurysm is characterised by intrinsic abnormalities within the vessel wall producing aortic dilatation at the level of the coronary sinus. It is an uncommon congenital abnormality affecting less than 0.1% of the population [2]. The clinical presentation varies, with most symptomatic patients presenting following sinus of Valsalva aneurysm rupture, with consequent manifestations reflecting the involved sinus, size of aneurysm and the site of decompression [3]. Other risks associated with sinus of Valsalva aneurysm include compression of adjacent cardiac structures and aortic regurgitation [4].

Sinus of Valsalva aneurysm most commonly involves the right coronary cusp, extending toward the right atrium and ventricle. In this case, the aneurysm extended into the coronary artery ostium. Coronary artery aneurysm, in contrast, is defined as coronary artery dilatation which exceeds the diameter of the largest normal coronary artery by 1.5 times. Coronary artery aneurysm is an uncommon clinical issue found in less than 5% of patients undergoing coronary angiography with giant coronary aneurysm considerably less frequent and defined as an artery greater than 2 cm in diameter and found in 0.02% of those undergoing coronary angiography [5, 6].

We report an unusual manifestation of sinus of Valsalva aneurysm involving the ostium of the right coronary artery producing an appearance typical of a giant coronary aneurysm. Relevant issues in terms of the management in an asymptomatic patient are discussed, as are the potential alternative causes of coronary artery aneurysm.

## 2. Case Report

**Figure 1:**
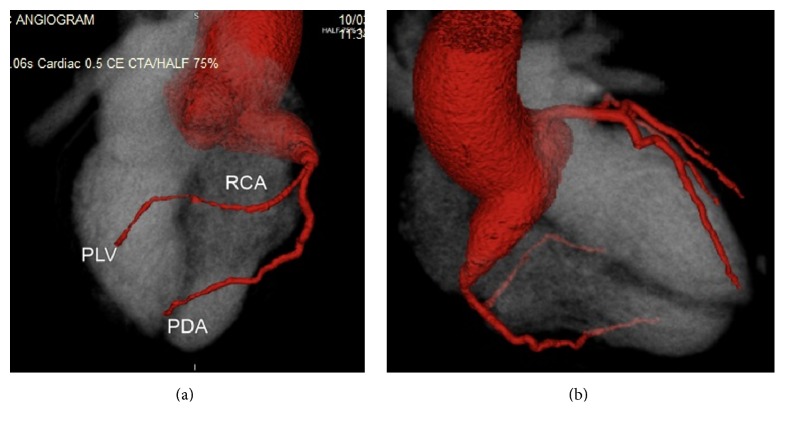
CT coronary angiography demonstrating right coronary sinus of Valsalva. Aneurysm viewed (a) anteriorly and (b) posteriorly.

**Figure 2:**
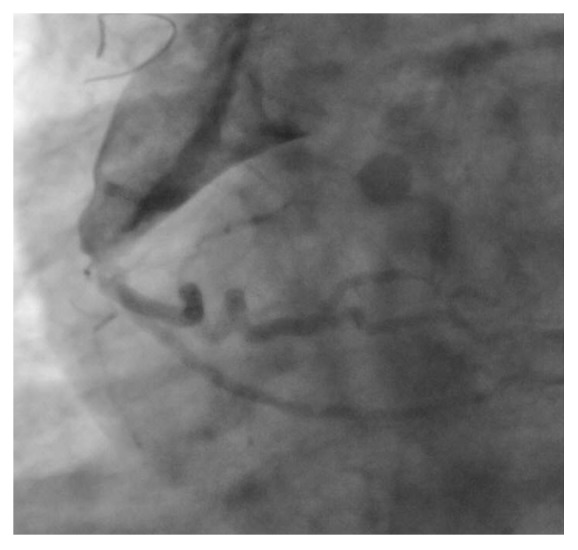
Coronary angiography of the right coronary sinus of Valsalva. The appearance was suggestive of aneurysmal dilatation of the right coronary artery ostium.

A 41-year-old Caucasian gentleman with a background of surgical closure of atrial septal defect aged 2 years old underwent routine echocardiography (surgical details unknown). The study was remarkable for the presence of a dilated right coronary ostium. No previous imaging was available for comparison, reflecting the remote nature of the patients' previous surgery. There was no history of collagen vascular diseases. There was no suggestion of an episode typical of Kawasaki disease in childhood. The patient was asymptomatic with preserved effort tolerance. Physical examination was normal. Specifically, there was no evidence of cardiac failure and no murmur was noted on examination. Transthoracic echocardiography demonstrated aortic root dilatation at the level of the sinus of Valsalva (4.9 cm) with an appearance suggestive of right coronary artery ostial dilatation; the proximal ascending aorta measured 4.1 cm. The remainder of the echocardiogram was within normal limits. There was no evidence of resting ischaemia or previous infarction. Given these findings, CT coronary angiography was performed which demonstrated dilatation involving the right sinus of Valsalva extending into the ostium of the right coronary artery (Figure 1). The remainder of his right coronary artery and the left sided coronary arteries were of normal calibre and had minor irregularities only noted. Subsequent diagnostic cardiac catheterisation confirmed the appearance of severe dilatation of the right coronary ostium (Figure 2). There was no obvious stenosis noted distal to the aneurysmal dilatation.

Due to concerns regarding possible subsequent coronary artery dissection, aneurysmal thrombus formation, and aneurysm rupture, the patient underwent surgical intervention.

Following redo sternotomy, a transverse aortotomy was performed. Inspection confirmed a right coronary sinus of Valsalva aneurysm involving the right coronary ostium. A bovine pericardial patch was placed between the aortic annulus and sinotubular junction excluding the aneurysm. The true ostium of the right coronary artery was then anastomosed to a saphenous vein graft; the internal mammary artery was of insufficient length to serve as a conduit. The postoperative course was uneventful.

**Figure 3:**
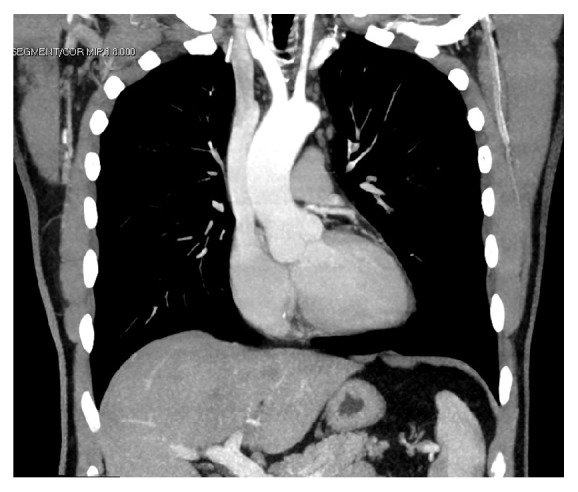
Postoperative contrast CT scan of the thorax demonstrating normal appearance of the right and left sinuses of Valsalva.

Postoperative echocardiography was within normal limits. Repeat CT imaging confirmed bypass graft patency and no residual right coronary ostial dilatation (Figure 3).

## 3. Discussion

Congenital sinus of Valsalva aneurysm results from intrinsic deficiencies of elastic and muscular tissue at the junction of aortic media and annulus fibrosus of aortic valve. This leads to focal dilatation producing a diverticulum in the involved coronary sinus [7]. The incidence is four times greater in males and more commonly affects people of Asian origin [8]. Sinus of Valsalva aneurysm typically occurs in the right sinus (75–90%), followed by noncoronary sinus (10–25%), with the remainder in the left coronary sinus [1]. Sundaram et al. established criteria to differentiate between acquired and congenital aneurysms, with acquired cases usually extending superiorly [9]. Sinus of Valsalva aneurysm is often associated with congenital defects particularly ventricular septal defect (30–50%), aortic insufficiency (20–30%), and bicuspid aortic valve (10%) and less commonly coarctation of the aorta, pulmonary stenosis, and atrial septal defects [10]. Sinus of Valsalva aneurysm may also complicate degenerative diseases (atherosclerosis, connective tissue disorders, or cystic medial necrosis) and infectious aetiologies, such as infective endocarditis, tuberculosis, and syphilis [2, 3].

When present, the appearance on imaging is typically of focal sinus dilatation adjacent to, rather than involving, the coronary ostia, producing a finger-like diverticulum [2, 7]. The aneurysm will typically extend toward either the right ventricle or right atrium, with extension into the ostium of the right coronary artery not previously reported.

In this case, the unusual site of the sinus of Valsalva aneurysm created an appearance suggestive of an ostial giant coronary artery aneurysm. Giant coronary artery aneurysm, which is defined as areas of coronary artery dilatation >2 cm, occur extremely infrequently. There are various potential aetiologies for the development of coronary artery aneurysms. Primary congenital anomalies of coronary arteries are rare, seen in less than 1% of patients undergoing diagnostic angiography, with the majority of coronary aneurysms being the result of atherosclerotic disease. Less common causes include Kawasaki disease and disorders of connective tissue in a manner similar to sinus of Valsalva aneurysm.

Clinical presentation, as in this case, may be asymptomatic prior to rupture and noted incidentally on imaging. Sinus of Valsalva aneurysms ruptures more frequently into the right ventricle and will typically produce symptoms, with the nature of symptoms depending on the site of rupture. Patients can present with chest pain, acute dyspnoea, palpitations, cardiogenic shock, or sudden death [11]. Once rupture has occurred, mean survival is 1 to 2 years. Death is usually due to congestive heart failure, but infective endocarditis has been reported as a cause of death in approximately 8% of cases [11].

Two-dimensional transthoracic echocardiography can detect up to 75% of all patients with sinus of Valsalva aneurysm [12]. The gold standard for diagnosis is cardiac catheterisation with aortography, and oximetry analysis may add to the diagnostic yield; other imaging techniques include MRI and CT angiography [13].

The decision to intervene on an asymptomatic, unruptured sinus of Valsalva aneurysm depends upon aneurysm size and involvement of adjacent structures. Rigorous blood pressure control is recommended for all patients, with surgical repair when aortic root diameter exceeds 5 cm [14]. The association with aortic incompetence may mandate aortic valve replacement in addition to aneurysm repair. The serious complications that may complicate rupture, combined with the low perioperative death rates, have triggered some authors to recommend operating on asymptomatic patients [15]. Surgical closure is either by simple suture or by patch closure of the sinus of Valsalva with the recurrence rate varying from 0% to 20% [16]. With regard to coronary artery aneurysm, the indications for the surgical treatment include severe coronary stenosis, fistula formation, compression of adjacent cardiac chambers, and rapid increase in aneurysm size [17].

The decision to proceed to surgical correction in cases of unruptured sinus of Valsalva aneurysm remains difficult. In this case, due to concerns regarding the possibility of ischaemic complications from the unusual involvement of the coronary ostia and as there was no serial imaging to compare rate of growth, it was felt that surgical correction to prevent subsequent complications was appropriate.
